# Assembly of coupled redox fuel cells using copper as electron acceptors to generate power and its *in-situ* retrieval

**DOI:** 10.1038/srep21059

**Published:** 2016-02-15

**Authors:** Hui-Min Zhang, Wei Xu, Gang Li, Zhan-Meng Liu, Zu-Cheng Wu, Bo-Geng Li

**Affiliations:** 1 Department of Environmental Engineering, Laboratory of Electrochemistry and Energy Storage, State Key Laboratory Clean Energy Utilization, Zhejiang University, Hangzhou 310027, China; 2 Institute of Environmental Engineering, East China Jiao Tong University, Nanchang, 330013, China; 3 State Key Laboratory of Chemical Engineering, School of Chemical and Biological Engineering, Zhejiang University, Hangzhou 310027, China

## Abstract

Energy extraction from waste has attracted much interest nowadays. Herein, a coupled redox fuel cell (CRFC) device using heavy metals, such as copper, as an electron acceptor is assembled to testify the recoveries of both electricity and the precious metal without energy consumption. In this study, a NaBH4-Cu(II) CRFC was employed as an example to retrieve copper from a dilute solution with self-electricity production. The properties of the CRFC have been characterized, and the open circuit voltage was 1.65 V with a maximum power density of 7.2 W m^−2^ at an initial Cu^2+^ concentration of 1,600 mg L^−1^ in the catholyte. 99.9% of the 400 mg L^−1^ copper was harvested after operation for 24 h, and the product formed on the cathode was identified as elemental copper. The CRFC demonstrated that useful chemicals were recovered and the electricity contained in the chemicals was produced in a self-powered retrieval process.

Heavy metal ions in industrial wastewater pose a potential threat to the living environment and human health due to their accumulation and enrichment *in vivo*^1^. Electrochemical approaches are especially effective for heavy metal removal but require a power supply^2^,^3^. Currently, instead of energy inputs, net energy extraction from waste has attracted considerable attention^4^,^5^,^6^,^7^. A metallurgical microbial fuel cell (MFC) is a successful example of copper recovery from wastewater combined with electricity generation^8^,^9^,^10^. However, MFC has some disadvantages, such as long start-up time, stringent working conditions, complicated operation and complex electron transfer mechanisms^11^. Despite increasing efficiencies in energy conversion and copper treatment due to reactor design and selection of operating parameters^9^,^12^, the MFCs can only be operated in neutral or near neutral media.

Fuel cells including solid oxide fuel cell^13^ are effective electrochemical devices that directly convert the chemical energy in fuels and oxidants into electrical energy^14^,^15^, and these fuel cells are a promising power supply. Inspired by fuel cells, we proposed a coupled redox fuel cell (CRFC) device that can successfully harvest energy and treat wastewater^16^,^17^. Thermodynamically, the anode redox potential of the CRFC should be lower than the cathode redox potential to ensure spontaneous electrochemical reactions. Using this approach, an interior bias can be produced to transfer the electrons of the anode to the cathode through the external circuit. In this cell, the low redox potential hydrogen-rich substances (or contaminants) in the aqueous solution act as fuels that can be oxidized on the catalytic anode to yield electrons. However, the high redox potential contaminants can function as electron acceptors that can be reduced on the cathode. Therefore, the chemical energy in contaminants can be converted to electrical energy, and some chemicals may be recovered. Therefore, the development of a suitable CRFC for use with certain industrial pollutants is an important area of research that could greatly extend the application of fuel cells in the field of environmental protection (or wastewater treatment), which would be economically beneficial.

Based on this hypothesis, a synthetic wastewater containing Cu^2+^ was chosen as the cathodic electron acceptor in this study. Copper, which is one of the universal heavy metals, widely exists in various industrial wastewaters from printed circuit board manufacturing as well as the electroplating, mining and metallurgical industries^18^. Copper is categorized as one of a priority pollutant by the US EPA (U.S. Environmental Protection Agency). However, Cu is a valuable industrial metal, and the recovery of copper from copper containing waste streams is attractive. To construct a high electromotive force to drive the electron transfer in a CRFC, an appropriate fuel is important. Sodium borohydride is a hydrogen-rich fuel used in the development of direct borohydride fuel cells (DBFCs)^19^,^20^,^21^. The BH_4_^−^ electrocatalytic reaction that occurs on the anode depends on the applied anode catalysts^19^,^22^,^23^. When Ni is used as the anode catalyst, the four-electron anode reaction and standard electrode potentials are shown in equation (1)^24^,^25^:


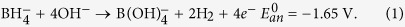


When Cu^2+^ was employed as an oxidant in the catholyte^8^, the pH of the catholyte was maintained at 3–4, which favors electroreduction of Cu^2+^. The final product would be Cu without the production of Cu_2_O or CuO. With pH control, only one reaction pathway becomes available for cathodic copper reduction, as shown by the following equation:


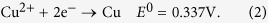


In the anode chamber, BH_4_^−^ is oxidized to B(OH)_4_^−^ with the generation of H_2_ and electrons. In the cathode chamber, Cu^2+^ is reduced to Cu. The overall theoretical electromotive force value is 1.987 V.

To control the pH in the cathode and prevent Cu^2+^ from catholyte being simultaneously transferred to the anode chamber, NaBH_4_ is maintained in an alkaline environment, and OH^−^ is prevented from diffusing into the cathode, which contains a four-chambered NaBH_4_-Cu CRFC separated by two cation exchange membranes (CEMs) and an anion exchange membrane (AEM), as shown in Fig. 1. When a current is generated by BH_4_^−^ electrooxidation in the anode chamber, electrons are transferred from the anode to the cathode through the external circuit. These electrons are captured by Cu^2+^ in the catholyte, which is reduced to Cu^0^. The positively charged Cu^2+^ species are prevented from leaving the cathode chamber by the AEM. However, negatively charged SO_4_^2−^ species move from the cathode chamber to the #3 chamber. Double CEMs filled with buffer solutions prevent small amounts of OH^−^ diffusion to the cathode chamber. Positive ions (i.e., Na^+^ or K^+^) are required in the buffer to maintain the ion balance. Therefore, positive ions can be transferred from the #2 chamber through the CEM to the #3 chamber. In the anode chamber, H_2_ is released into solution or the air, resulting in excess positively charged species moving from the #1 chamber to the #2 buffer district. The product of Cu^2+^ reduction is deposited on the cathode surface along with the generation of electricity. To determine the feasibility of copper recovery, Cu^2+^ electrochemical deposition at the cathode with simultaneous production of electricity was investigated using a NaBH_4_-Cu CRFC. In addition, this study focused on characterizing the ability of catalytically oxidative electrodes to remove metal ions and developing a process for the useful recovery of chemicals. The performance of the CRFC was characterized in terms of its power generation, Cu^2+^ removal efficiency and cathodic efficiency as well as the composition of the deposited products on the cathode under different Cu^2+^ concentrations.

## Results and Discussion

### Selection of fuel and effect of fuel concentration

To a certain extent, the open circuit voltage (OCV) may account for the fuel cell production capacity^14^ (i.e., high OCV would produce a higher output voltage). Due to the inevitable polarization loss from a cell, a low OCV would result in a working cell with low efficiency and affect the pollutant removal. To obtain a high OCV, different systems are measured by introducing different compositions of fuels into the anode chamber and a copper ion solution in the cathode chamber. The results are shown in Table 1. When using urea, ethanol, glucose, starch and sodium borohydride as fuels and copper ions as the oxidant, the obtained OCVs are 0.58 V, 0.65 V, 0.78 V, 0.65 V and 1.54 V, respectively, which indicate that all these fuels can be used for copper removal with the production of electricity. In addition, when sodium borohydride was used as the fuel, the OCV of the NaBH_4_-Cu CRFC was significantly higher than that for Cu CRFCs using other fuels. When 0.2 M NaBH_4_ and 1 M KOH were used as the fuel along with a cathode copper ion concentration of 400 mg L^−1^, an open circuit voltage of 1.54 V was produced. Therefore, to obtain a higher output voltage, sodium borohydride was selected as the fuel to investigate copper removal. In addition, KOH was selected as the supporting electrolyte because sodium borohydride is relatively stable in alkaline solutions.

**Figure 2:**
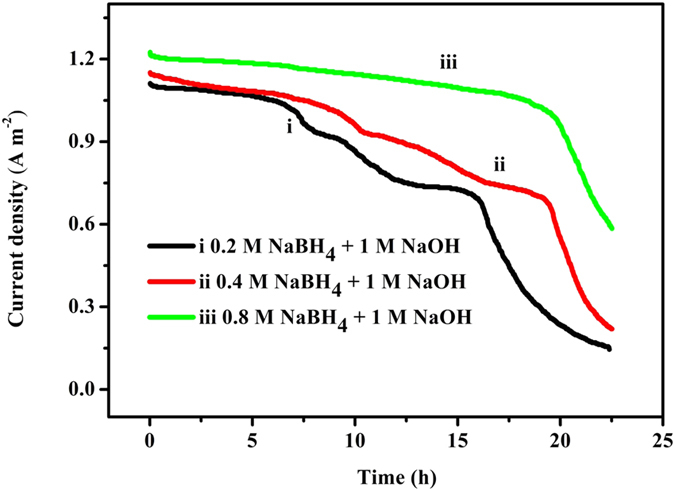
Current evolution as a function of time for the NaBH_4_-Cu(II) CRFC. Anolyte: 0.2 M NaBH_4_ (i, black), 0.4 M NaBH_4_ (ii, red) and 0.8 M NaBH_4_ (iii, green) in 1 M NaOH; Catholyte: 800 mg L^−1^ Cu^2+^.

Figure 2 shows the current evolution as a function of time using different initial NaBH_4_ levels. The OCV increased from 1.54 V to 1.67 V as NaBH_4_ increased from 0.2 M to 0.8 M. The initial current density for all three levels was 1.11, 1.13 and 1.22 A m^−2^ after adding an external load of 1000 Ω. A maximum difference of only 0.11 A m^−2^ was observed in the beginning. Then, the maximum difference (difference between the current densities at high concentration and low concentration) increased to 0.39 A m^−2^ at 16 h and further increased to 0.72 A m^−2^ at 20 h. This result indicates that excess fuel can maintain a high current output. However, in the NaBH_4_ fuel cell, high concentrations (1.5–2.4 M) of sodium borohydride decreased the electron transfer number and increased fuel hydrolysis, which can result in low fuel efficiency^25^. Therefore, a low NaBH_4_ concentration of 0.2–0.8 M was used as the fuel in the NaBH_4_-Cu(II) CRFC.

In addition, the current output of the three groups with different fuel concentrations exhibited a similar pattern. Above a certain value, the current output was stable after the external load of 1000 Ω followed by a turning point that appeared near 15 h, 18 h and 20 h, for the three levels. Then, the current decreased sharply in nearly a straight line. Near these turning points, copper in the catholyte is still detected, which indicates that the fuel was nearly consumed at these turning points.

### Effect of initial Cu^2+^ concentration

**Figure 3:**
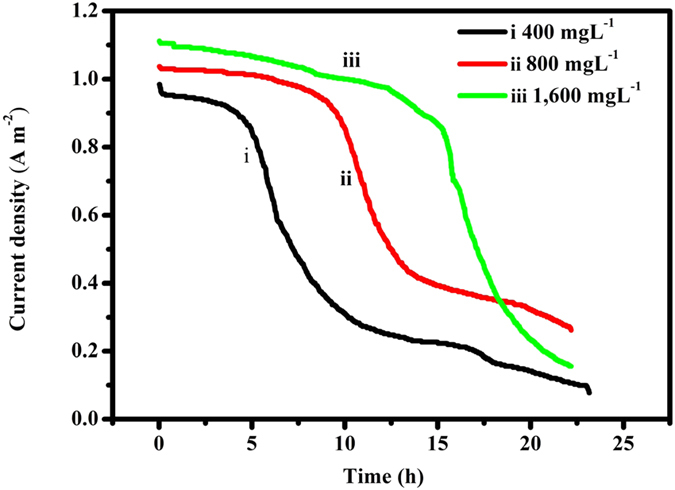
Current evolution as a function of time for the NaBH_4_-Cu(II) CRFC. 1,600 mg L^−1^ Cu^2+^ (iii, green), 800 mg L^−1^ Cu^2+^ (ii, red) and 400 mg L^−1^ Cu^2+^ (i, black); Anolyte: 0.2 M NaBH_4_ in 1 M NaOH.

The results in Fig. 3 indicate that the NaBH_4_-Cu(II) CRFC exhibits a considerable electrical output for various initial Cu^2+^ concentrations in the catholyte. When 1,600 mg L^−1^ Cu^2+^ was employed as the electron acceptors, the CRFC reached an OCV of 1.65 V and an initial current density of 1.11 A m^−2^ with a constant external load of 1000 Ω. As the initial Cu^2+^ concentration decreased to 800 mg L^−1^, an OCV of 1.59 V was achieved, and the corresponding initial current density decreased to 1.05 A m^−2^. A further decrease in the initial Cu^2+^ concentration to 400 mg L^−1^ resulted in an OCV and initial current density of 1.54 V and 0.98 A m^−2^, respectively.

The current production at all of initial copper levels as a function of time is shown in Fig. 3. Interestingly, the current density curves exhibited the same trend and reached a plateau at the beginning and then decreased quickly. Notably, the plateau became larger as the initial Cu^2+^ concentration increased. When the Cu^2+^ concentration was increased to 1,600 mg L^−1^, the plateau lasted approximately 15 h at a high level. Then, the current density decreased gradually in the following 10 h after the 15-h plateau. This result was reasonable because a higher copper concentration (1,600 mg L^−1^) can provide more electron acceptors that allow the cell to run longer at a high current density compared to a lower copper level (400 mg L^−1^). It was also noticed that the current density from 1,600 mg L^−1^ Cu^2+^ deteriorated to lower than that from 800 mg L^−1^ Cu^2+^ after 18 hrs running in Fig. 3. The reason for this may be that more fuels needed to be consumed for 1,600 mg L^−1^ Cu^2+^ and so that the P_max_ dropped faster than that for 800 mg L^−^1 Cu^2+^ against 0.2 M NaBH_4_.

The voltage under a load of 1000 Ω with an initial 800 mg/L Cu^2+^ was 720 mV, which was higher than that of the microbial desalination cell (568 mV)^12^. This result may be due to two factors. First, the theoretical anodic potential was −1.35 V ([OH^−^]  = 1 M, [BH_4_^−^] = 2.0 M) using NaBH_4_ as the fuel in this study, which was much more negative than −0.296 V (pH = 7, [CH_3_COO_3_^−^] = [HCO_3_^−^] = 0.05 M) for the acetate fuel used in their work^12^. Secondly, the internal resistance of the NaBH_4_-Cu(II) CRFC with an initial Cu^2+^ concentration of 800 mg L^−1^ was only 18.7 Ω, which was significantly less than that of the MFC (41.6 Ω)^12^. A low internal resistance indicates low voltage losses and a high output voltage.

**Figure 4:**
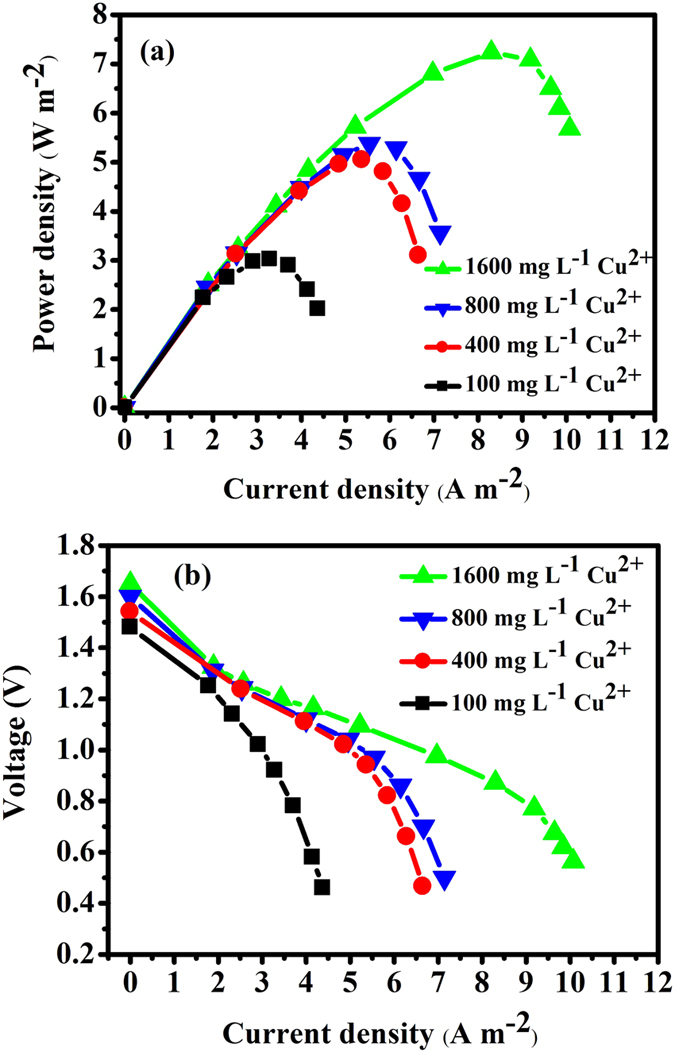
Power density (**a**) and polarization (**b**) curves for the NaBH_4_-Cu(II) CRFC. 1,600 mg L^−1^ Cu^2+^ ((▲), 800 mg L^−1^ Cu^2+^ (▼), 400 mg L^−1^ Cu^2+^ (●) and 100 mg L^−1^ Cu^2+^ (■)); Anolyte: 0.2 M NaBH_4_ in 1 M NaOH.

Figure 4 shows the polarization curves for the maximum electricity generation using the procedure in the methods section. An increase in the initial Cu^2+^ concentration from 100 mg L^−1^ to 1,600 mg L^−1^ results in an increase in the maximum power density (P_max_) by a factor of 2.4 from 3.0 W m^−2^ at 0.82 V, 3.2 A m^−2^ to 7.2 W m^−2^ at 0.92 V, 8.5 A m^−2^. The P_max_ data indicates that when higher initial Cu^2+^ concentrations were used, a higher power density at a higher current density was obtained.

### Effect of temperature

**Figure 5:**
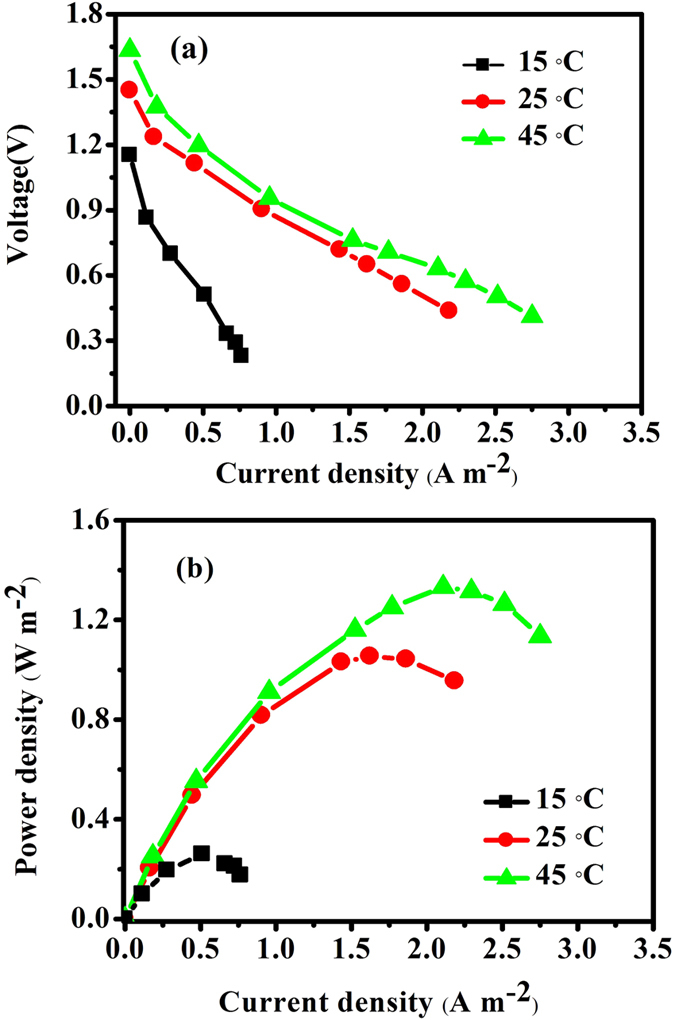
Polarization curve (**a**) and power profiles (**b**) at different temperatures. (15 °C (■), 25 °C (●), 45 °C (▲) and fixing the anolyte at 0. 2 M NaBH_4_ in 1 M NaOH and catholyte at 400 mg L^−1^ Cu^2+^ in 0.5 M HAC-NaAC (pH = 3.5).

The results in Fig. 5 demonstrate the effect of temperature on the voltage and power density. As shown in Fig. 5(a), the power density and voltage output increase as the temperature increased. This result is due to two factors. First, a high temperature can accelerate the electrochemical reaction and reduce the activation resistance. Secondly, an increase in the temperature is favorable for mass transfer and decreases concentration polarization. As shown in Fig. 5(b), a P_max_ of 0.26 W m^−2^ at a current density of 0.5 A m^−2^ at 15 °C was observed. When the operation temperature increased from 15 °C to 25 °C, an increase in P_max_ by a factor 2.1 was observed (i.e., from 0.5 W m^−2^ to 1.05 W m^−2^). A further increase in temperature to 45 °C results in a P_max_ of 1.33 W m^−2^, which is a factor of 1.3 higher than 1.05 W m^−2^ at 25 °C. In addition, a high temperature would accelerate fuel hydrolysis and reduce fuel efficiency. Therefore, in this study, the operation temperature was determined to be 25 °C.

### Copper removal

Table 2 shows the copper removal percent (R_Cu_) and cathodic efficiency (η_cath_) of the NaBH_4_-Cu(II) CRFC at three initial copper levels. The copper removal rate was 75.08 ± 0.3% at 16 h for an initial copper concentration of 400 mg L^−1^, and an increase in the time to 24 h can result in an increase in the removal percentage to 99.9 ± 0.4%. In addition, the cathodic efficiency was ~100%. However, no more than 100% was observed at 24 h when the initial copper concentration was increased to 1,600 mg L^−1^, which indicates that more electrons were recorded as current being produced than those consumed by copper electroreduction. Therefore, another oxidant was present in the catholyte and consumed a portion of the electrons released from the anode. Oxygen is most likely present in the catholyte, which would result in the production of a background current due to oxygen reduction. This phenomenon easily occurred at a low copper concentration due to the standard potential of oxygen (1.23 V vs SHE) being significantly greater than the copper reduction potential (0.337 V vs SHE). Nevertheless, when a high copper concentration was present in the catholyte, this background current can be ignored because the trace oxygen amount was much smaller than the copper concentration.

### Product of the process

**Figure 6:**
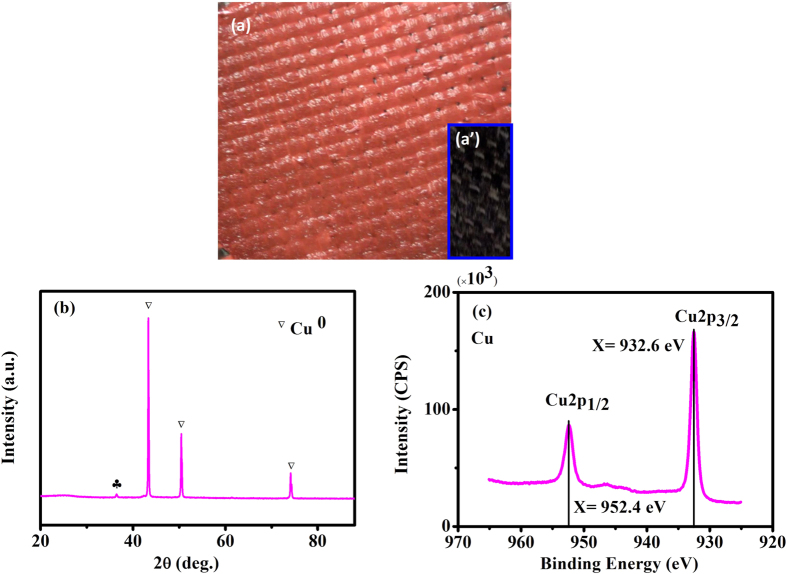
Cathodic reduction of copper. Photo of copper deposited on a carbon cloth electrode (**a**) compared to the initial electrode (a’), XRD (**b**), XPS (**c**).

The oxidation state of the deposited Cu species has been identified. A brick red product (see Fig. 6(a)) was formed on the cathode. XRD and XPS measurements were employed to determine the composition and oxidation states of the reduction species. The diffraction peaks at 2θ values of approximately 43.3°, 50.4° and 74.1° in the XRD (Fig. 6(b)) can be indexed to copper. Figure 6(c) shows the typical XPS spectra of the Cu species. For all of the deposited species, the binding energies of Cu 2p3/2 and Cu 2p1/2 were 932.4 eV and 952.2 eV, respectively. The Cu 2p3/2 peak can be used to determine the oxidation states of the Cu species. The position of the main peak of Cu 2p3/2 at approximately 932.6 eV corresponded to elemental Cu. However, no Cu(II) and Cu(I) were detected. Cu(II) deposition in the catholyte bulk was not observed due to the low pH of the bulk solution (pH <3.5). Therefore, copper can be removed from the aqueous solution using this NaBH_4_-Cu(II) CRFC system.

### Feature and ability of the CRFC

Self-driven copper retrieval from solutions containing Cu^2+^ combined with power harvesting makes this new approach suitable for copper recovery. The efficiencies are comparable to the results achieved with other electrochemical copper recovery methods, such as electrowinning^27^,^28^. In contrast to the electrowinning requiring power supply, which consumed 3.3 kWh kg^−1^ Cu^28^, the CRFC produced power, which was equivalent to an energy production of 9.3 kWh Kg^−1^ of Cu (data derived from 800 mg L^−1^ Cu^2+^ as the catholyte). This produced energy may allow this process to be applied more widely.

Another feature of the CRFC is its ability to generate electricity. The comparison between CRFC and MFCs will certainly be unavoidable even though the mechanism of electricity generation from the two processes is completely different. For example, by applying an initial 800 mg L^−1^ Cu^2+^ concentration and an external resistance of 1000 Ω, the initial current density in the CRFC is ca. 1.05 A m^−2^, which is 3 times higher than the peak current density of the MFC (<0.3 A m^−2^) using ions bridge composed of membranes and salt solutions^12^ as the separator. At the same initial 800 mg L^−1^ Cu^2+^concentration, NaBH4-Cu CRFC produce a P_max_ of 5.4 W m^−2^, which is 24, 22 and 7 times the results reported by An *et al.* (0.226 W m^−2^)^12^, Tao *et al.* (ca. 0.25 W m^−3^)^10^ and Ter Heijne *et al.* (0.8 W m^−2^)^8^, respectively. Cheng *et al.*^9^ and Motos *et al.*^26^ substantially improved the power density and achieved ca. 2.0 W m^−2^, which is still less. More than 99.9% removal of 400 mg L^−1^ Cu^2+^ was achieved in this study in 24 h. In contrast, 80 h was needed in the MFC study^12^, and 30 h was required by the CRFC to achieve 99.6% removal of 800 mg L^−1^ Cu^2+^. In addition, 168 h was need for 99.88% removal of 1000 mg L^−1^ in a MFC^8^ because it is time-consuming and laborious to transmit electrons from the interior of microbial cells to the surface of electrodes^5^,^11^. Overall, the new assembly consisting of a coupled redox fuel cell using copper as an electron acceptor combines the production of electricity with *in situ* retrieval of copper.

## Conclusions

Considerable recoveries of both electricity and copper metal without energy consumption can be successfully achieved in a coupled redox fuel cell (CRFC) device. Hydrogen used as a fuel in sodium borohydride in an alkaline medium in this system would result in a high OCV. The generation of electricity will increase as the initial Cu^2+^ concentration increases. An increase from 0.2 M to 0.8 M in the initial NaBH_4_ concentration favors current production from 1.11 to 1.22 A m^−2^ at the initial stage, and from 0.70 to 1.08 A m^−2^ at 16 h. Increase in temperature to 45 °C results in a P_max_ of 1.33 W m^−2^, which is significantly higher than P_max_ of 0.26 W m^−2^ at 15 °C. Elemental copper was uniformly formed on the cathode to provide a unique electrowinning process for copper recovery that was self-powered.

## Methods

### NaBH_4_-Cu(II) CRFC assembly

The NaBH4-Cu CRFC reactor contained four chambers named #1, #2, #3 and #4. Liquid was not pumped through the chambers but was standing still. The copper sulfate solution in 0.5 M HAC-NaAC (pH = 3.5) was fed as the electron acceptor into the cathode chamber (#4). 0.2 M NaBH_4_ in a 1 M NaOH solution was used as the fuel in the anode chamber (#1) except fuel effect experiments. In the NaBH_4_-Cu CRFC, the pH values in the four chambers were maintained using a buffer solution (i.e., 7 (#2), 3.5 (#3) and 3.5 (#4)) and an alkaline solution (#1). #4 is separated from the other three parts by one AEM. One CEM is next to the anode chamber, which separate #1 from #2. This chamber functions as a selective bridge, permitting the passage of positively charged sodium ions but not negatively charged hydroxyl ions. A CEM is used to separate the two sections (#2 and #3) and further consolidates the role of the previous CEM. The #2 and #3 chambers were filled with buffer solution containing 0.5 M PBS (KH_2_PO_4_-Na_2_HPO_4_, pH 7) and 0.5 M HAC-NaAC (pH = 3.5).

Ni/C catalysts were prepared according to a previously published method^29^. Then, Ni/C was mixed with deionized water (0.83 μL/mg Ni/C), 5% Nafion dispersion (6.67 μL/mg Ni/C) and isopropyl alcohol (3.33 μL/mg Ni/C). After ultrasonic dispersion for 30 min, this mixture was painted on a carbon fiber cloth (Tansu, Jinlin, China) with a loading of 5 mg Ni/cm^−2^ 29. The resulting cloth was used as the anode in the NaBH_4_-KOH solution after it was dried in air for 24 h. The same area carbon cloth was used as the cathode.

### Analyses and calculations

Concentrations of the Cu(II) samples at the interval time were measured using the AAS (Atomic absorption spectroscopy) method. The composition and oxidation states of the cathodic product was measured by XRD (X-ray diffraction) using Shimadzu XRD-6000 with a Cu-Kα radiation source and XPS (X-ray photoelectron spectroscopy) using ESCALAB_250Xi with an Al-Kα excitation source, respectively. A data acquisition system (PISO-813, ICP DAS Co., Ltd) was employed to record the voltage (U) every minute. Polarization curves, current and power density were obtained according to a previously published protocol^16^. The Cu (II) removal efficiency (R_cu_) was calculated based on the Cu (II) concentrations at time t and the initial time using R_cu_ = (C_0_-C_t_)/C_0_. The amount of copper reduction was calculated based on the difference between Cu (II) reduction obtained in the control experiment (C_0_-C_con_) and that obtained under a closed circuit (C_0_-C_t_). This value was divided by the electricity production by integrating the current over time, which is defined as the cathodic efficiency (*η*_cath_) and calculated as follows:


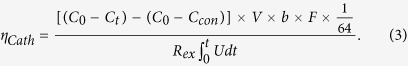


where *η*_*cath*_ is the cathodic efficiency (%), *t* is the operation time (h), *R*_*ex*_ is the constant external load (R_ex_ = 1000 Ω) under a closed circuit, *C*_*t*_*, C*_*0*_ is the Cu (II ) concentration (mg L^−1^) at time *t* and the initial time, respectively, *C*_*con*_ is the Cu (II ) concentration (mg L^−1^)) at time *t* under the control experiment, *V* is the volume of the catholyte (20 mL), *b* is the number electrons of per mol Cu (II) to copper (b = 2 mol e^−^/mol Cu (II)) and *F* is the Faraday constant (96485 C mol^−1^).

## Additional Information

**How to cite this article**: Zhang, H.-M. *et al.* Assembly of coupled redox fuel cells using copper as electron acceptors to generate power and its *in-situ* retrieval. *Sci. Rep.* **6**, 21059; doi: 10.1038/srep21059 (2016).

## Figures and Tables

**Figure 1:**
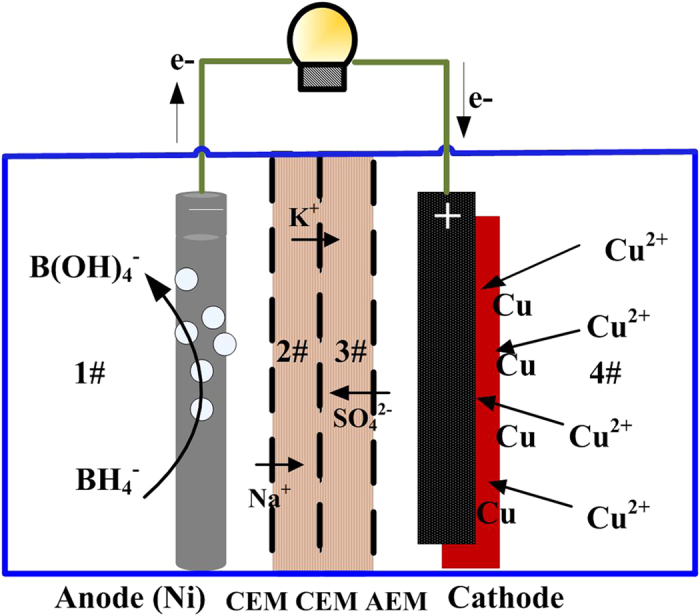
Schematic diagram of NaBH4-Cu(II) CRFC.

**Table 1:** OCVs using different fuels in the anolyte.

Catholyte	Anode catalyst	Fuel components	OCV (V)	Standard emf (V)*
400 mg L^−1^Cu^2+^	5 mg Ni/cm^2^	0.33 M Urea + 1 M KOH	0.56	1.097
		1 M Ethanol + 1 M KOH	0.62	1.077
		0.2 M Glucose + 1 M KOH	0.75	1.577
		1% Starch	0.64	—
		0.2 M NaBH_4_ + 1 M KOH	1.54	1.987

^*^Standard electromotive force (emf): Standard potential of the Cu^2+^/Cu redox pair minus the standard potential of the fuel redox pair in the anode.

**Table 2:** Cu^2+^ removal rate (R_Cu_) and cathodic efficiency (η_cath_) under different initial copper concentrations.

Cu(II) C_0_(mgL^−1^)	16 h		24 h	
	R_cu_(%)	η_cath_(%)	R_cu_(%)	η_cath_(%)
400	75.08 ± 0.3	90.6 ± 0.4	99.9 ± 0.4	108.2 ± 0.3
800	65.3 ± 0.4	92.3 ± 0.3	85.6 ± 0.6	99.3 ± 0.4
1,600	34.6 ± 0.5	79.6 ± 0.6	46.2 ± 0.3	92.6 ± 0.5
